# Supplementary material for: Stage-specific GATA3 induction promotes ILC2 development after lineage commitment
Source: Nat Commun. 2024 Jul 5;15:5610. doi: 10.1038/s41467-024-49881-y (PMC11226602; doi:10.1038/s41467-024-49881-y)
Supplement: Supplementary file 1 — Supplementary Information [file 41467_2024_49881_MOESM1_ESM.pdf]

Supplementary Information

Stage-specific GATA3 induction promotes ILC2 development after lineage commitment

Hiroki Furuya[1,†], Yosuke Toda[1,†], Arifumi Iwata[1,†,*], Mizuki Kanai[1], Kodai Kato[1], Takashi Kumagai[1], Takahiro Kageyama[1], Shigeru Tanaka[1], Lisa Fujimura[2], Akemi Sakamoto[2,3], Masahiko Hatano[2,3], Akira Suto[1], Kotaro Suzuki[1], and Hiroshi Nakajima[1,4,*]

[1]Department of Allergy and Clinical Immunology, Graduate School of Medicine, Chiba University.
[2]Biomedical Research Center, Chiba University.
[3]Department of Biomedical Science, Graduate School of Medicine, Chiba University.
[4]Chiba University Synergy Institute for Futuristic Mucosal Vaccine Research and Development (cSIMVa), Chiba, Japan

[†]These authors contributed equally to this work.
[*]Corresponding authors: Drs. Arifumi Iwata (aiwata@chiba-u.jp) and Hiroshi Nakajima (nakajimh@faculty.chiba-u.jp)

This Supplementary Information file contains:

Supplementary Figure 1-12
Supplementary Table 1, 2

Supplementary Figure 1

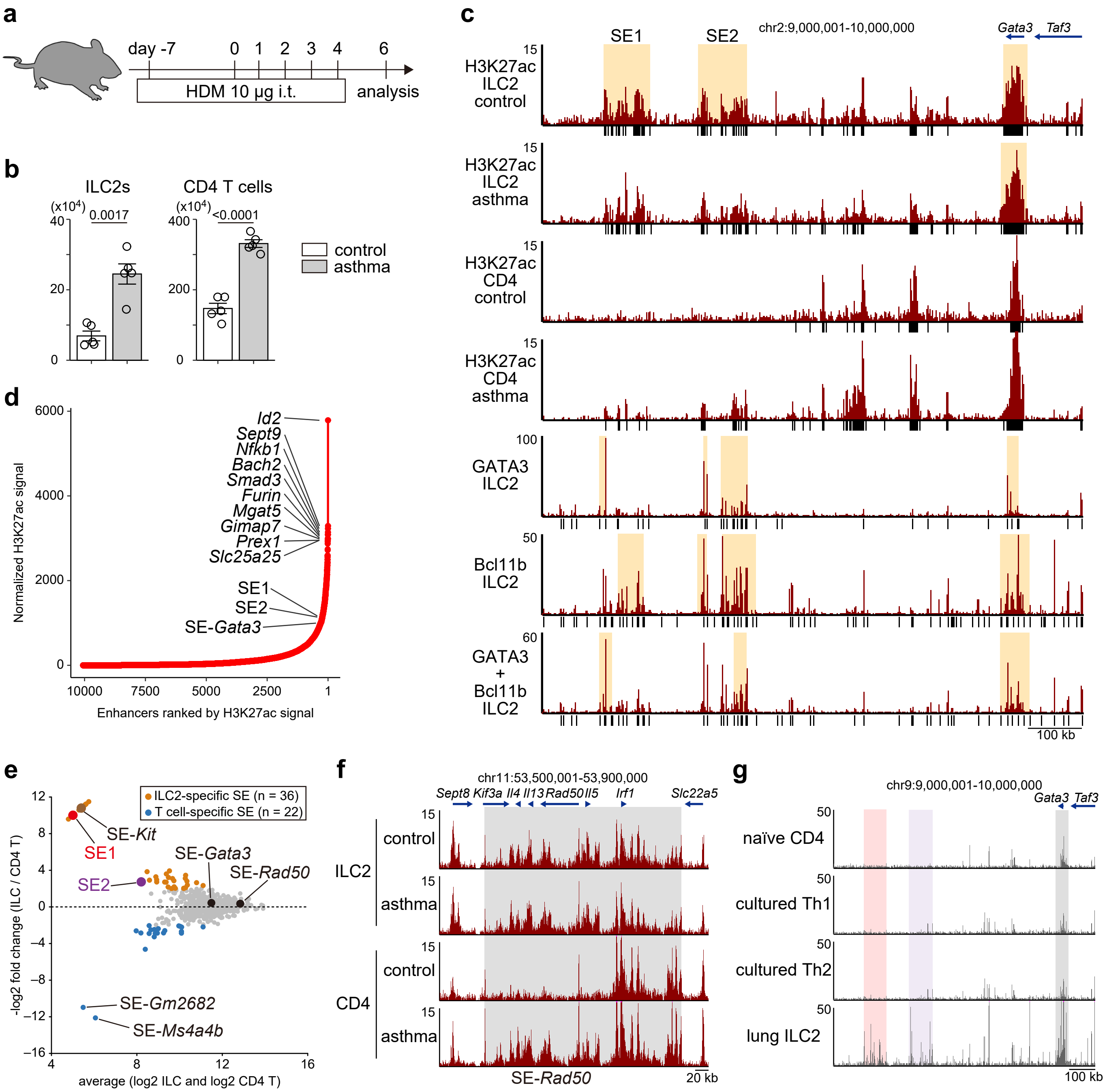

Supplementary Figure 1. ILC2-specific super-enhancers identified via H3K27ac ChIP-seq. **a** Experimental procedure of HDM-induced allergic airway inflammation. C57BL/6J mice were intratracheally administered with house dust mites (HDM) on days -7, 0, 1, 2, 3, 4. i.t.: intratracheal. **b-f** Samples were harvested from control mice and asthma mice on day 0 and day 6. ILC2 ($CD45^{+}Lin1^{-}Thy1.2^{+}ST2^{+}$ cells) and CD4 T cells ($CD45^{+}Lin2^{-}CD4^{+}$ cells) were isolated. Lin1: CD4, CD5, CD8a, CD11b, CD11c, CD19, CD31, CD49b, FcεR1, TCRβ, TCRγδ, Ter119; Lin2: CD8a, CD11c, CD49b, TCRγδ, EpCAM. **b** The number of ILC2s and CD4 T cells in the lung, n=5. Data are presented as mean±SE. Statistical analysis was performed using unpaired, two-sided Welch's *t*-test. p values are shown on the graphs. c ILC2s and CD4 T cells were subjected to ChIP-seq analysis of H3K27 acetylation (H3K27ac). UCSC tracks of ChIP-seq of ILC2 and CD4 T cells, and GATA3 (GSE11187), Bcl11b (GSE131082), GATA3+Bcl11b ChIP-seq data. Black bars indicate typical enhancers, and orange boxes indicate super-enhancers. **d** The plot of H3K27ac signal versus rank of stitched enhancers in control ILC2. **e** MA-plot of tag counts on the super-enhancers based on H3K27ac in ILC2 and CD4 T cells under HDM-asthmatic conditions. Total: 624 SEs. A fold change > 4 was considered specific. **f** UCSC tracks of H3K27ac ChIP-seq for ILC2 and CD4 T cells in the *Rad50-Irf1* region. Rectangles indicate super-enhancer regions. **g** UCSC tracks of ATAC-seq for naïve CD4 T cells, cultured Th1 cells, cultured Th2 cells (GSE159505), and lung ILC2 in steady-state conditions. Source data are provided as a Source Data file.

Supplementary Figure 2

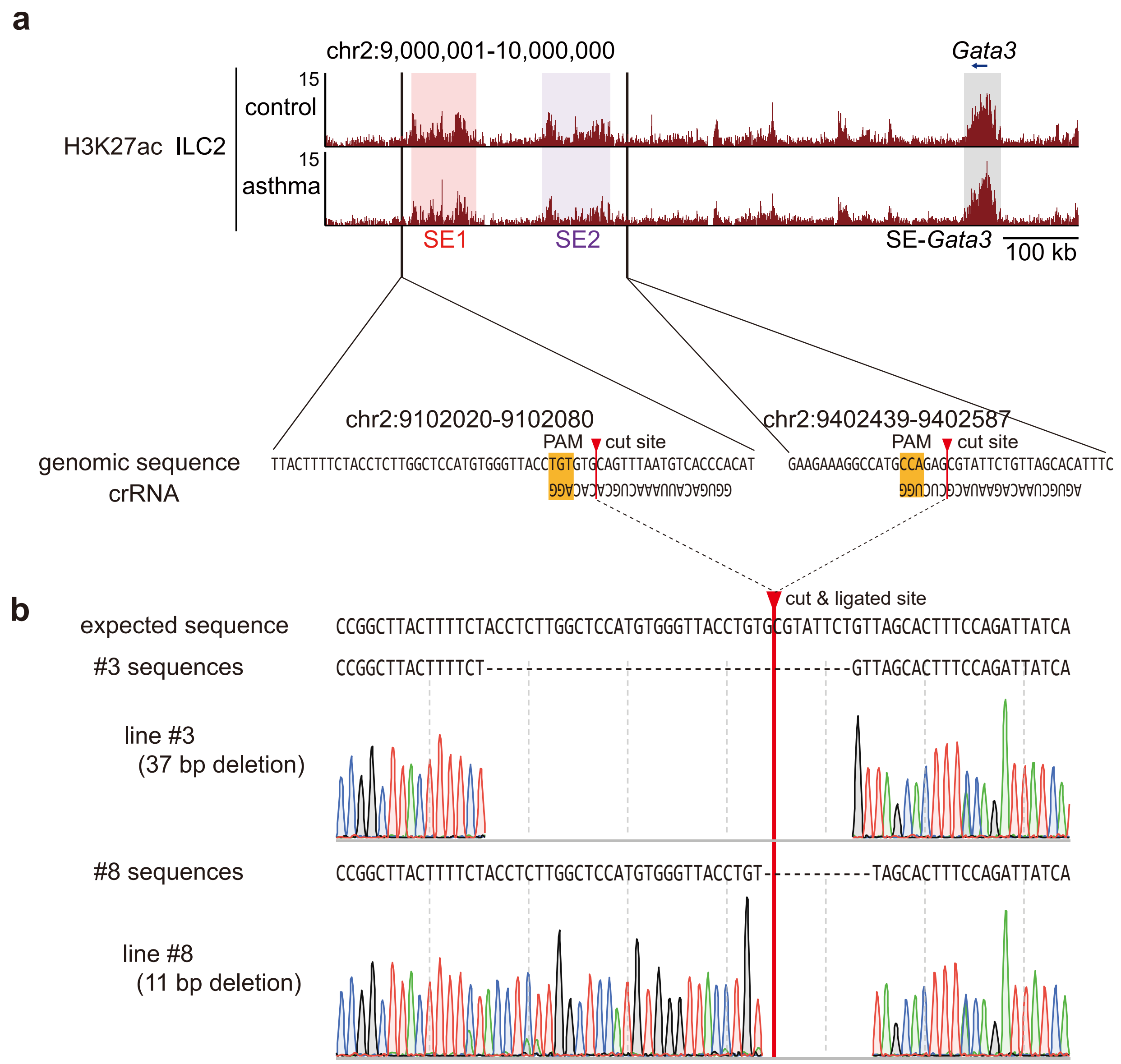

Supplementary Figure 2. Generation of G3SEKO mice.
a Upper panel: UCSC genome tracks around the *Gata3* and G3SE region. Lower panel: Genomic sequence of upstream/downstream crRNA target regions. **b** DNA sequences of G3SE deleted loci from two lines of generated G3SEKO mice.

Supplementary Figure 3

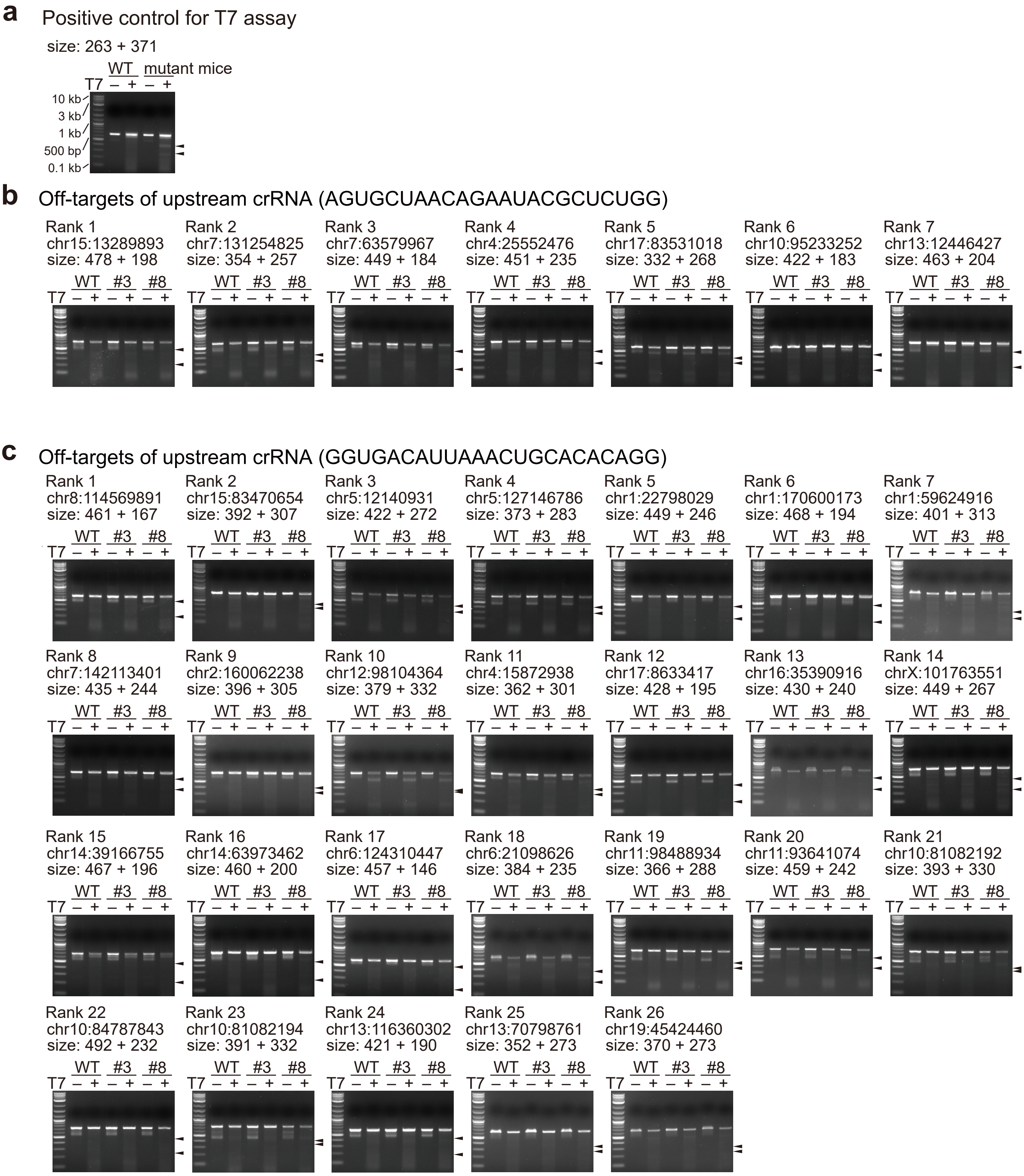

Supplementary Figure 3. Off-target analyses of crRNA-derived cleavage.
The off-target candidates induced by crRNA were identified using CAS-OFFinder. The tail DNA from wild-type and G3SEKO mice was mixed in equimolar ratios to generate PCR templates. The regions flanking the candidate sites were amplified by PCR, and the off-target insertion/deletion was determined using the T7 Endonuclease mismatch detection method. Arrowheads indicate the predicted cleavage sites. **a** The positive control of T7 endonuclease mismatch detection method. Template DNA was extracted from the tail of mutant mice generated by single crRNA. **b-c** T7 assay results for the off-target candidates of upstream (**b**) and downstream (**c**) crRNAs. Uncropped gel photos are provided as a Source Data file.

Supplementary Figure 4

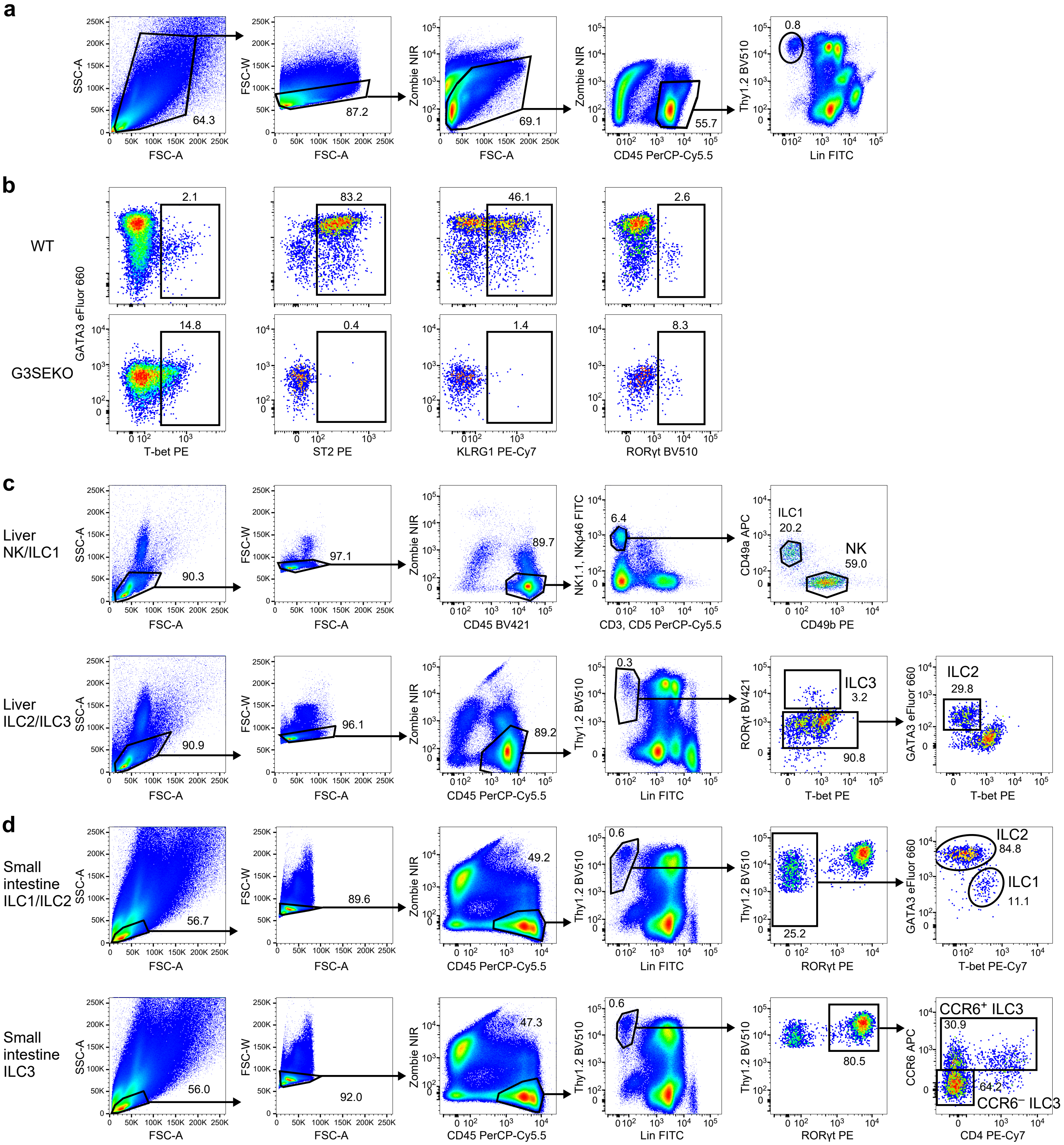

Supplementary Figure 4. Gating strategies for lung, liver, and small intestinal ILCs.
a-b Gating strategy for lung ILCs (**a**) and ILC2 (**b**). Lin: CD3ε, CD4, CD5, CD8a, CD11b, CD11c, CD19, CD49b, Gr1, B220, Ter119. **c** Gating strategy for liver NK cells and ILC1 (upper panels) and ILC2 and ILC3 (lower panels). Lin: CD3ε, CD4, CD5, CD8a, CD11b, CD11c, CD19, Gr1, B220, Ter119. **d** Gating strategy for small intestinal ILC1/ILC2 (upper panel) and ILC3 (lower panel). Lin: CD3ε, CD5, CD8a, CD11b, CD11c, CD19, Gr1, B220, Ter119.

Supplementary Figure 5

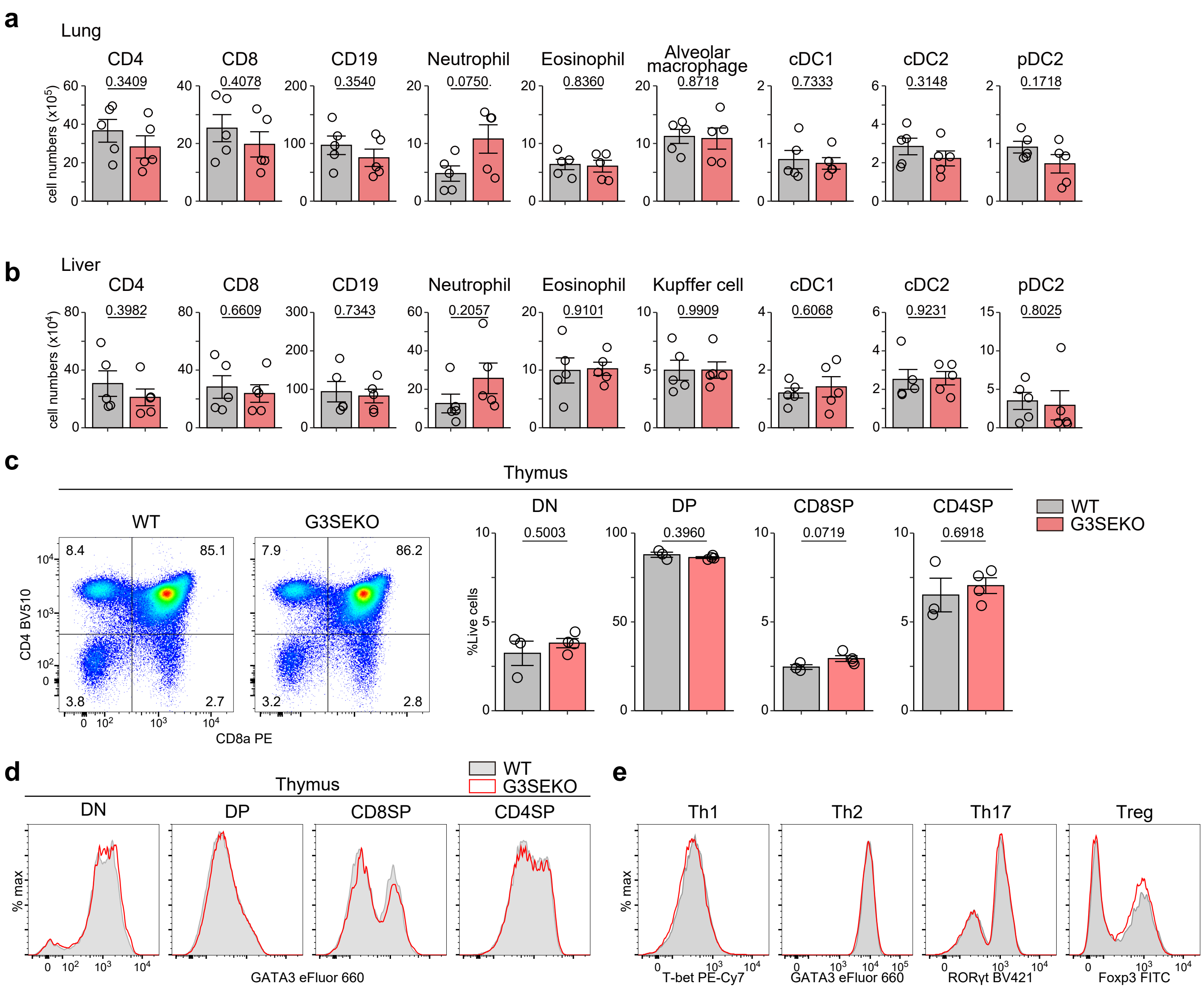

Supplementary Figure 5. Immune cell development and helper T cell differentiation in G3SEKO mice. **a-b** Distribution of lymphocytes and myeloid populations in the lung (**a**) and the liver (**b**) of WT and G3SEKO mice. n=5. **c** Flow cytometry analysis of thymocytes in WT (n=3) and G3SEKO (n=4) mice. DN: double negative, DP: double positive, SP: single positive. **d** GATA3 expression in thymocytes. **e** Naïve CD4 T cells were cultured under T cell subset-polarizing conditions for three days, and master transcription factor expression was analyzed. (**a-c**), Each data point indicates one mouse from two independent experiments. Data are presented as mean±SE. Statistical analysis was performed using unpaired, two-sided Welch's t-test. p values are shown on the graphs. (**d-e**) Shown are representative of six WT mice and five G3SEKO mice from two independent experiments. Source data are provided as a Source Data file.

Supplementary Figure 6

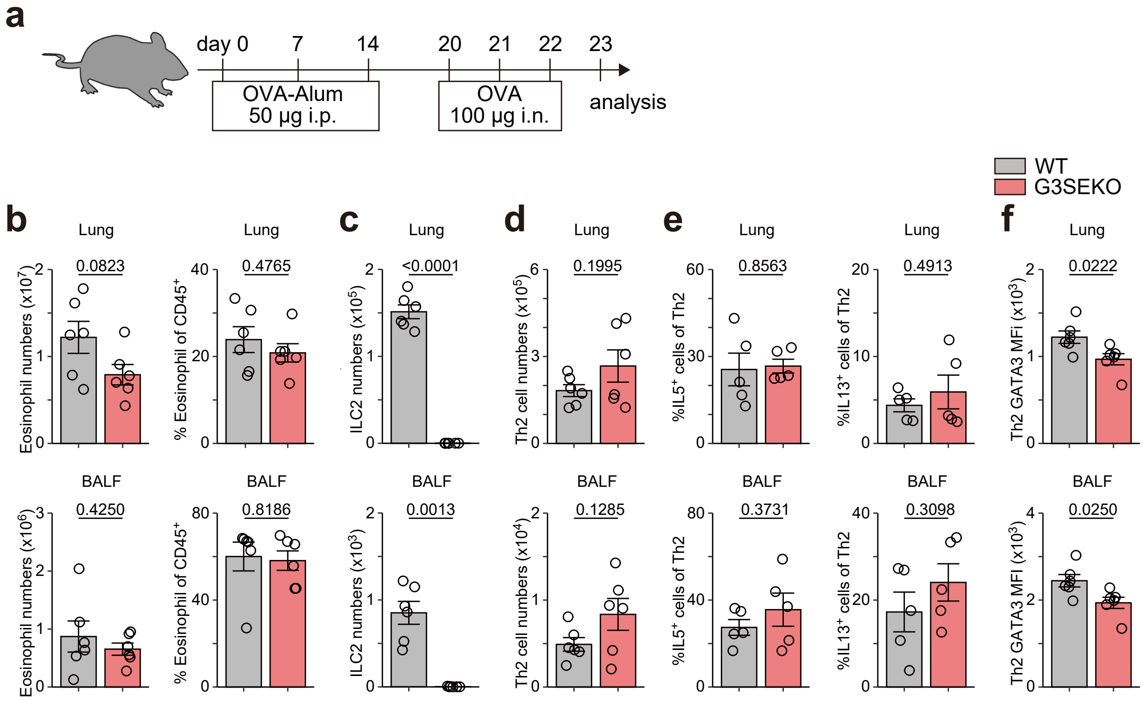

Supplementary Figure 6. OVA-induced allergic airway inflammation in G3SEKO mice.
a Scheme of OVA-induced asthma model. **b** Eosinophil inflammation in lung and bronchoalveolar lavage fluid (BALF). **c** Cell numbers of ILC2s ($Lin^-Thy1.2^+ST2^+$ cells). **d** Cell numbers of Th2 cells ($CD3\varepsilon^+CD4^+ST2^+$ cells). **e** IL-5 and IL-13 expression of Th2 cells. **f** GATA3 expression of Th2 cells. **b-f** Each data point indicates one mouse from three (**b-d, f**, n=6) or two (**e**, n=5) independent experiments. Data are presented as mean±SE. Statistical analysis was performed using unpaired, two-sided Welch's t-test. p values are shown on the graphs. Source Data are provided as a Source Data file.

Supplementary Figure 7

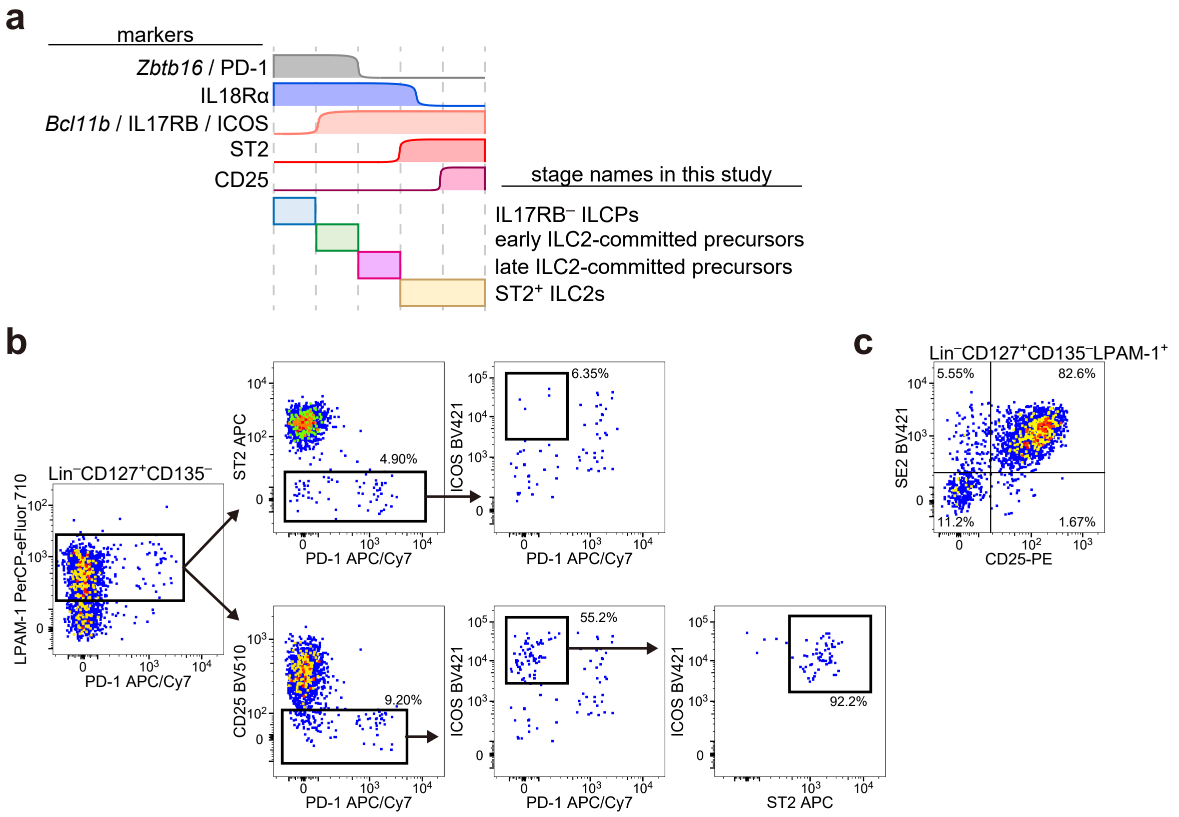

Supplementary Figure 7. ILC2 lineages in the BM.

a The terms of ILC2 lineages in the BM in this study. **b** Flow cytometry analysis of WT BM cells for the late ILC2-committed precursors. **c** Flow cytometry analysis of ST2 and CD25 of WT Lin−CD127+CD135−LPAM-1+ BM cells. The plots are representative of two independent experiments (**b-c**).

Supplementary Figure 8

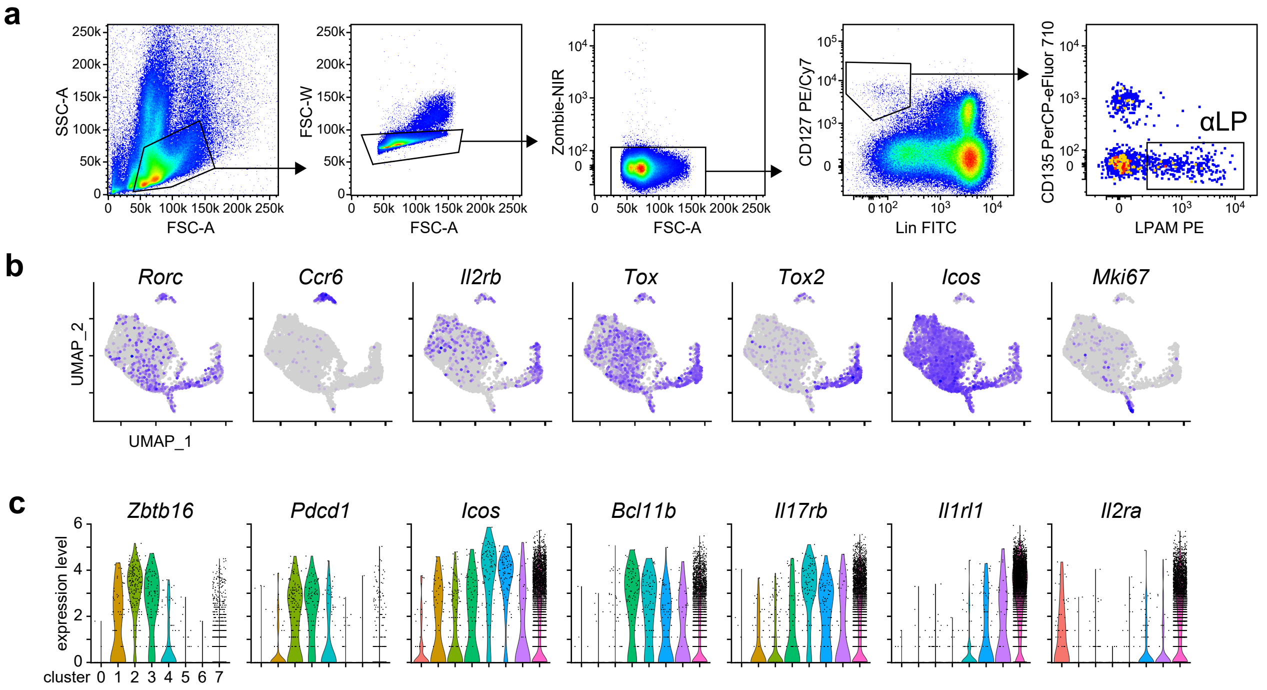

Supplementary Figure 8. Gating strategies and the expression of ILC-related genes.
a Gating strategy of αLP-sorting for scRNA-seq experiments. Lin: CD3ε, CD4, CD5, CD8a, CD11b, CD11c, CD19, CD49b, Gr1, B220, Ter119. **b-c** Feature plots (**b**) and violin plots (**c**) show the expression of ILC development-related genes.

Supplementary Figure 9

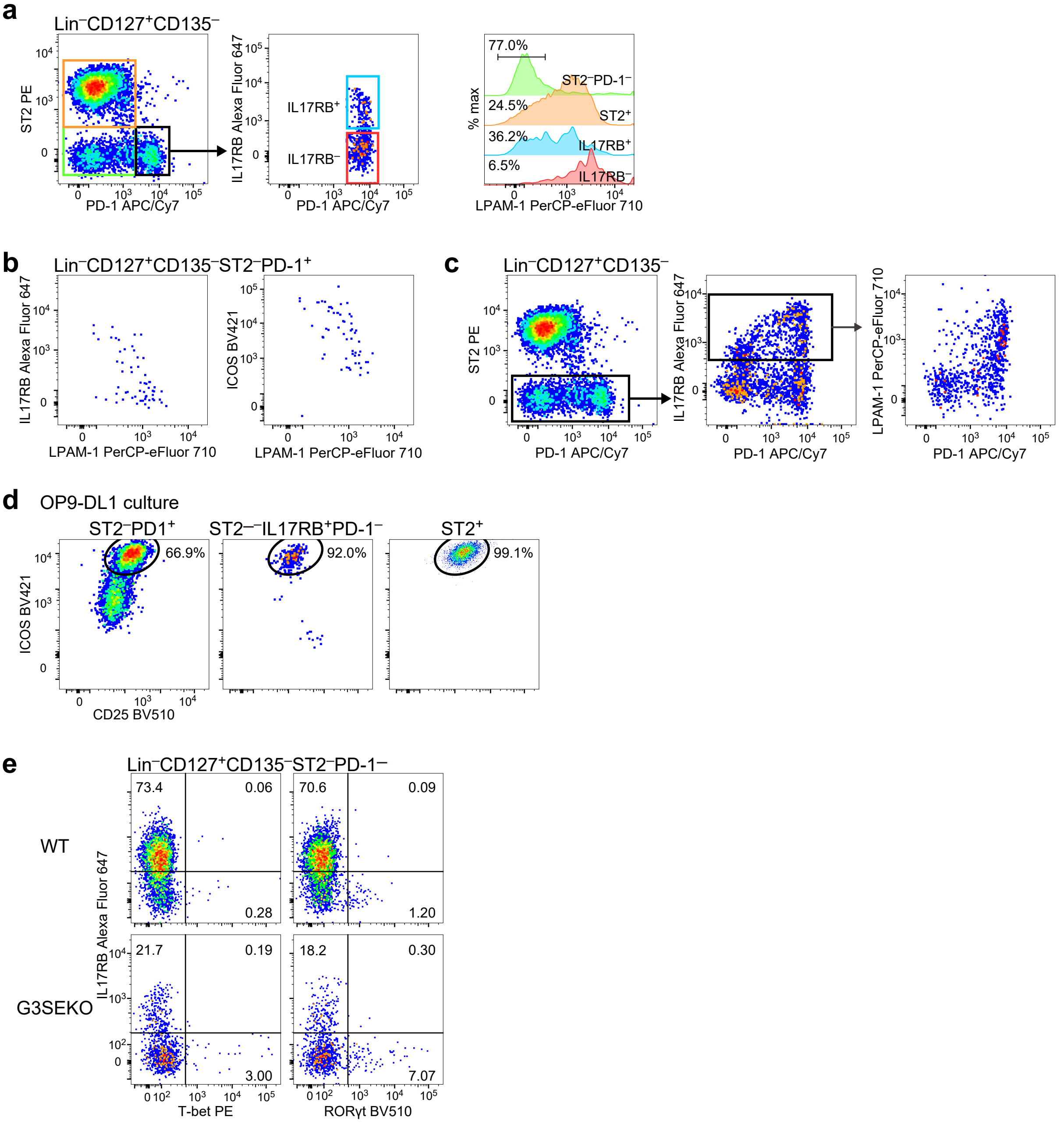

Supplementary Figure 9. Analysis of late ILC2-committed precursors in the BM.
a Flow cytometry analyses of LPAM expression in Lin$^-$CD127$^+$CD135$^-$ cells in WT BM. **b** Relationship between LPAM-1 expression and IL17RB or ICOS expression in WT PD-1$^+$ ILCPs. **c** Relationship between PD-1 expression and LPAM-1 expression in WT ST2$^-$IL17RB$^+$ cells. **d** Indicated cells were sorted from the WT Lin$^-$CD127$^+$CD135$^-$ BM cells and were cultured on OP9-DL1 cells in the presence of IL-7 and SCF for 5 days. Flow cytometry analysis of cultured cells. **e** Flow cytometry analyses of Lin$^-$CD127$^+$ST2$^-$PD-1$^-$ BM cells. Lin: CD3ε, CD4, CD5, CD8a, CD11b, CD11c, CD19, CD49b, CD135, Gr1, B220, Ter119. The plots were representative of four (**a-d**) and two (**e**) mice from two independent experiments.

Supplementary Figure 10

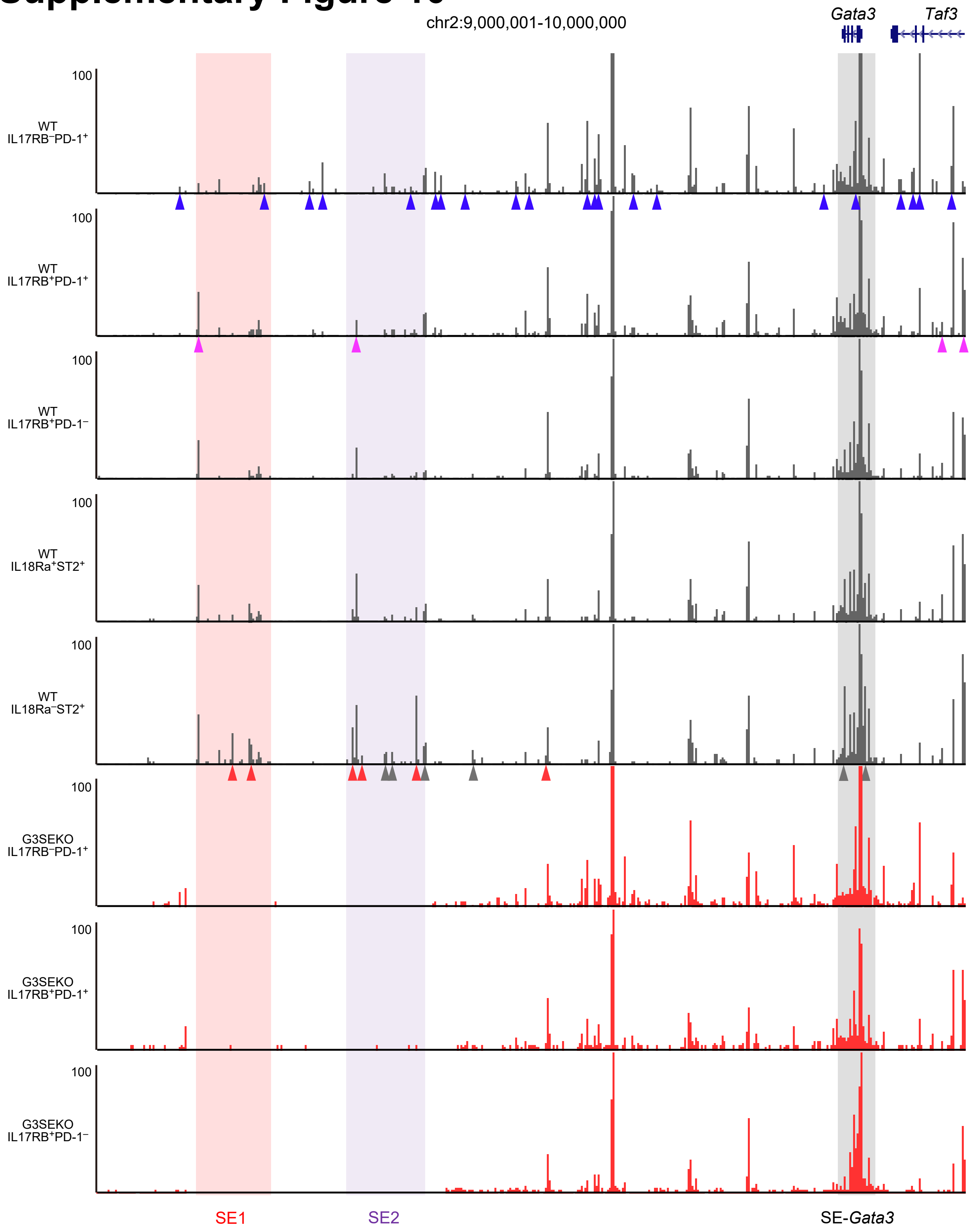

Supplementary Figure 10. ATAC-seq of ILC2 lineages in the BM of WT and G3SEKO mice. ATAC-seq was performed for IL17RB$^-$ ILCP, early and late ILC2-committed precursors, IL18Rα$^+$ST2$^+$ ILC2 and IL18Rα$^-$ST2$^+$ ILC2 from WT BM cells and IL17RB$^-$ ILCP, early and late ILC2-committed precursors from G3SEKO BM cells. Red, purple, and grey arrowheads indicate open chromatin regions; red arrow heads indicate late opened chromatin, purple arrowheads indicate early opened chromatin, and blue arrowheads indicate closed chromatin during ILC2 development.

Supplementary Figure 11

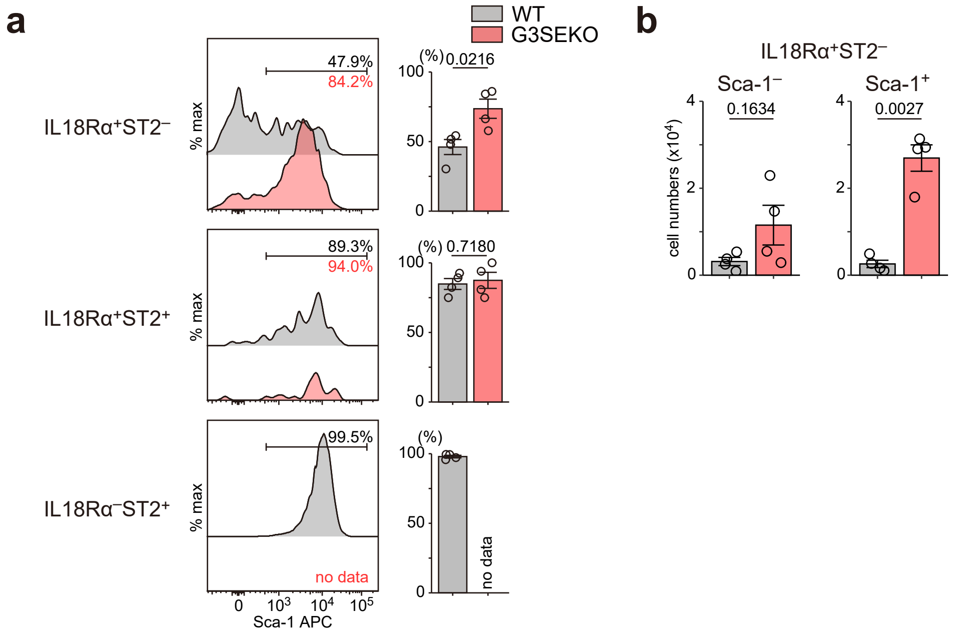

Supplementary Figure 11. Sca-1 expression of lung ILC2 lineages.

a, Sca-1 expression of indicated cells in the lung. n=4. Data were representative of four mice from three independent experiments. **b**, Cell numbers of IL18Rα^{+}ST2^{-}Sca-1^{-} cells and IL18Rα^{+}ST2^{-}Sca-1^{+} cells. (**a**, **b**) Data are presented as mean±SE. Statistical analysis was performed using unpaired, two-sided Welch's t-test. p values are shown on the graphs. Source data are provided as a Source Data file.

Supplementary Figure 12

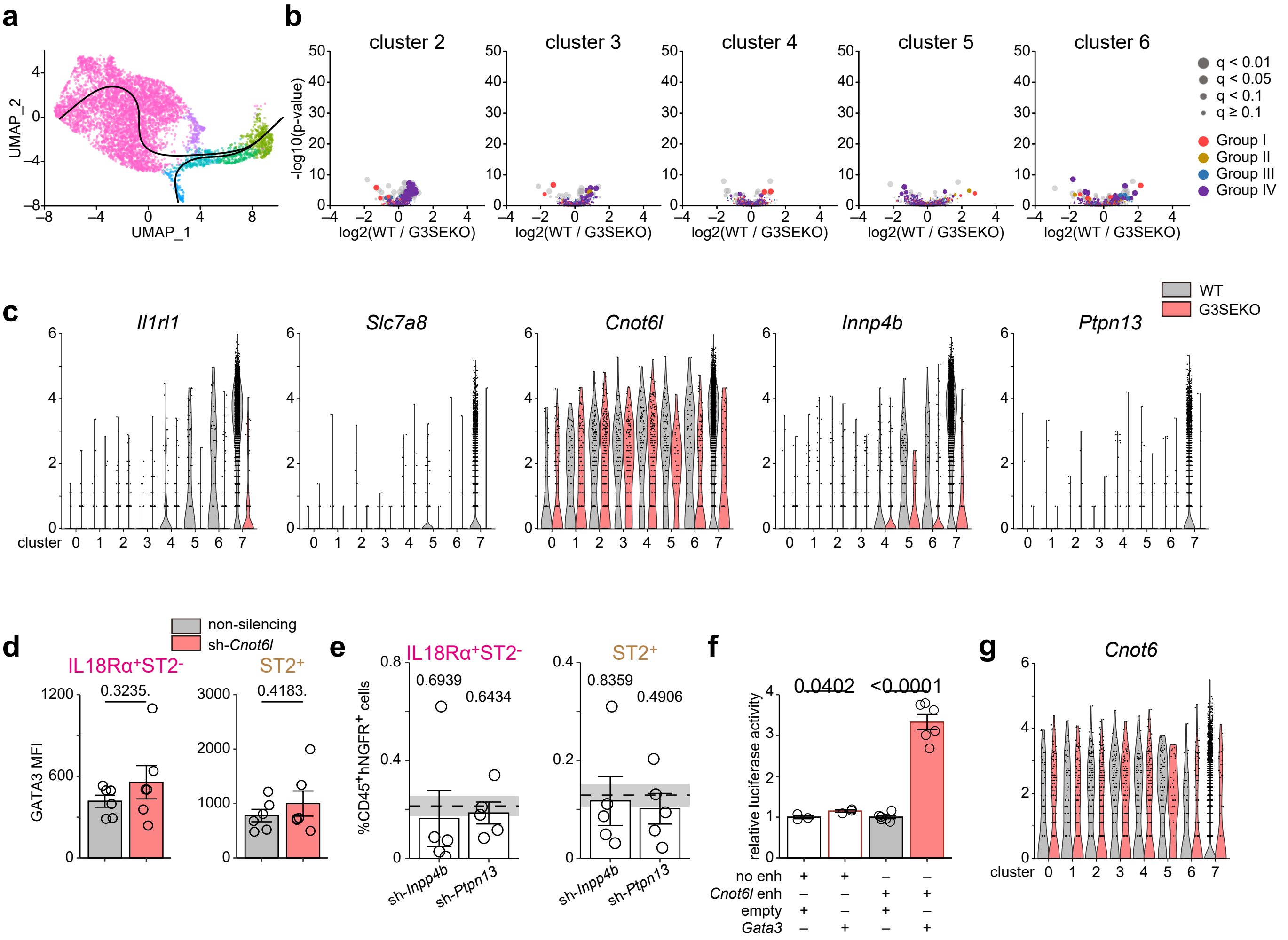

Supplementary Figure 12. Supporting data related to Fig. 5.

a Pseudotime analysis for clusters 2-7 by slingshot. **b** Differentially expressed genes between WT and G3SEKO cells in cluster 2 to cluster 6. **c** Expression of the candidate genes, related to Fig. 5f. **d** Gata3 expression of sh-*Cnot6l* transduced IL18Rα$^+$ST2$^-$ cells and ST2$^+$ cells in the lung, related to Fig. 5h. n=6. **e** Lung ILC frequency (CD45$^+$hNGFR$^+$Lin$^-$Thy1.2$^+$ cells) in CD45$^+$hNGFR$^+$ cells, related to Fig. 5h. Dash lines and grey areas indicate the mean and SE of non-silencing samples, respectively. Statistical analysis were performed between indicated shRNA-transduced cells and non-silencing vector-transduced cells. n=5. **f** Dual-luciferase Reporter Assay for the *Cnot6l* enhancer region in 293T cells transiently transduced with MSCV-empty vector or MSCV *Gata3* vector. no enhancer: n=3, *Cnot6l* enhancer n=6. **g** Expression of *Cnot6* by scRNA-seq analysis. Each data point indicates one mouse from four (**d, e**) and two (**f**) independent experiments. (**d-f**) Data are presented as mean±SE. Statistical analysis was performed using unpaired, two-sided Welch's t-test. p values are shown on the graphs. Source data are provided as a Source Data file.

Supplementary Table 1. The list of oligonucleotides

name	sequence
G3SEKO 5' crRNA	Alt-R1/AGUGCUAACAGAAUACGCUC/Alt-R2
G3SEKO 3' crRNA	Alt-R1/GGUGACAUUAAACUGCACAC/Alt-R2
G3SEKO genotype forward primer	GACCCTTTAGTTTAGCTCCTTGC
G3SEKO genotype reverse primer	CTCACCATGGAAACTTACAGCTC
G3SEWT genotype forward primer	GCTGGATAGAAGGCTGACTCATA
G3SEWT genotype reverse primer	ATGGGGTACAATGCTAAACAGAA
G3SE2KO 5' crRNA	Alt-R1/AGUGCUAACAGAAUACGCUC/Alt-R2
G3SE2KO 3' crRNA	Alt-R1/GUGGAGGUGGUAUACCCAAC/Alt-R2
G3SE2KO genotype forward primer	TATTCTCCCTTGTGGTCAATCC
G3SE2KO genotype reverse primer	CAATGTCTCCACTATGCTCCAG
G3SE2WT genotype forward primer	GCTGGAGTGGTAAGGAGCAG
G3SE2WT genotype reverse primer	GGAGCCTTGAGTCCTGAGTG
miR-30 common forward primer	CAGAAGGCTCGAGAAGGTATATTGCTGTTGACAGTGAGCG
miR-30 common reverse primer	CTAAAGTAGCCCCTTGAATTCCGAGGCAGTAGGCA
shRNA targeting *Ptpn13*	TGCTGTTGACAGTGAGCGTTGCTACCAGAAATCGATACTAT AGTGAAGCCACAGATGTATAGTATCGATTTCTGGTAGCACT GCCTACTGCCTCGGA
shRNA targeting *Sptssa*	TGCTGTTGACAGTGAGCGCGCATTTGCACGTTGTGATAAAT AGTGAAGCCACAGATGTATTTATCACAACGTGCAAATGCAT GCCTACTGCCTCGGA
shRNA targeting *Cnot6l*	TGCTGTTGACAGTGAGCGATGGAAACAGAGCAATACTTTAT AGTGAAGCCACAGATGTATAAAGTATTGCTCTGTTTCCACT GCCTACTGCCTCGGA
shRNA targeting *Inpp4b*	TGCTGTTGACAGTGAGCGTAACTGCATTATCGCTAAAGTAT AGTGAAGCCACAGATGTATACTTTAGCGATAATGCAGTTCT GCCTACTGCCTCGGA
shRNA targeting *Tespa1*	TGCTGTTGACAGTGAGCGAATCTCGTATCCCTGCAAGATTT AGTGAAGCCACAGATGTAAATCTTGCAGGGATACGAGATGT GCCTACTGCCTCGGA
ATAC-seq universal primer Ad1	AATGATACGGCGACCACCGAGATCTACACTCGTCGGCAGC GTCAGATGTG
ATAC-seq index primer Ad2.1	CAAGCAGAAGACGGCATACGAGATTCGCCTTAGTCTCGTG GGCTCGGAGATGT
ATAC-seq index primer Ad2.2	CAAGCAGAAGACGGCATACGAGATCTAGTACGGTCTCGTG GGCTCGGAGATGT
ATAC-seq index primer Ad2.3	CAAGCAGAAGACGGCATACGAGATTTCTGCCTGTCTCGTGG GCTCGGAGATGT
ATAC-seq index primer Ad2.4	CAAGCAGAAGACGGCATACGAGATGCTCAGGAGTCTCGTG GGCTCGGAGATGT
ATAC-seq index primer Ad2.5	CAAGCAGAAGACGGCATACGAGATAGGAGTCCGTCTCGTG GGCTCGGAGATGT
Off-target_upstream_Rank1_F	TTTGGAGGTTTGGCTTCCTA
Off-target_upstream_Rank1_R	ATCAAAGTGGACCTGGTTGC
Off-target_upstream_Rank2_F	TGTCCAATGGCAGACTTCAA
Off-target_upstream_Rank2_R	GGTGGTCAGTGTCCATCAGA
Off-target_upstream_Rank3_F	CATAGGGGACTTTCGGGATAA
Off-target_upstream_Rank3_R	TCACTGGGTGTCCCAACTAA
Off-target_upstream_Rank4_F	GGCAGTAGATTCCTGGGATG
Off-target_upstream_Rank4_R	TGCTACCGCTGAAAAAGGTC
Off-target_upstream_Rank5_F	TGAGGTTCCCCATCACTACG
Off-target_upstream_Rank5_R	GAGACGTGTTGCATCAAGGA
Off-target_upstream_Rank6_F	AGAATCGAAGTTGGCACACC
Off-target_upstream_Rank6_R	ACCCCATCTCCCCAATTAAC
Off-target_upstream_Rank7_F	CAGTGCCAGTTCCAAGACAG

Off-target_upstream_Rank7_R	TGTAGCCCCAAGGAATTCTG
Off-target_downstream_Rank1_F	ATGTGTGCGTGTGGATGAGT
Off-target_downstream_Rank1_R	TGGGATCTAGGCATCTGGAG
Off-target_downstream_Rank2_F	AAGCCCAAGTTCAGTCCTCA
Off-target_downstream_Rank2_R	AGGTCCCCTCTGTCACCTTT
Off-target_downstream_Rank3_F	GTGTTGGGGGAGTGGATAGA
Off-target_downstream_Rank3_R	TGTCAACAGAGCCACTTTCG
Off-target_downstream_Rank4_F	GTGGCATGTGCCATCTGTAG
Off-target_downstream_Rank4_R	ATTTCCAGTCAGCGGTTGTC
Off-target_downstream_Rank5_F	CTTCATCAGGGACTGCTCCT
Off-target_downstream_Rank5_R	CCCCCTGCCTCAACTTATTT
Off-target_downstream_Rank6_F	GAGCTGCCTCTGCAAACTCT
Off-target_downstream_Rank6_R	AACTCTGGGGCATCTGATTG
Off-target_downstream_Rank7_F	GGCTCACAACCACCCATAAC
Off-target_downstream_Rank7_R	AAGGCACATGGATGAAAAGG
Off-target_downstream_Rank8_F	CCTGTGTGTGTATGCGTGTG
Off-target_downstream_Rank8_R	GGGGGTTCCAGTGTACAGAA
Off-target_downstream_Rank9_F	CCACTCGTCCCACGTACTTT
Off-target_downstream_Rank9_R	TGGGTGCGTCTAAAGTGTGA
Off-target_downstream_Rank10_F	TTCTGTGCCCTTTTGCTCTT
Off-target_downstream_Rank10_R	CCCCAAATCCACTTCTCTCA
Off-target_downstream_Rank11_F	CCCTCCCTCCCTGTTTTCTA
Off-target_downstream_Rank11_R	TTCATCCCACCTGCTTTTTC
Off-target_downstream_Rank12_F	CGAGGGAAGGAATGTTTGAA
Off-target_downstream_Rank12_R	TATGCACGGCTAGTCAGCAC
Off-target_downstream_Rank13_F	TGGCTCAGTGCATAAAGGTG
Off-target_downstream_Rank13_R	TTTTGTCTTTCTCCCCGAAA
Off-target_downstream_Rank14_F	CCAACTGGCAGATTCCCTTA
Off-target_downstream_Rank14_R	GGGATCTGACACCGTCATCT
Off-target_downstream_Rank15_F	TCTGTGCGAGTGTGTGAGTG
Off-target_downstream_Rank15_R	GAAGCATCCTGAGCCCATTA
Off-target_downstream_Rank16_F	GCTCAGCTCAGCTACCTGCT
Off-target_downstream_Rank16_R	CCCTATTTGTCCTGCTCTGC
Off-target_downstream_Rank17_F	TGTCACCGTCCTTTTTAGCC
Off-target_downstream_Rank17_R	TAGCACTGTTTGGGGAGGT
Off-target_downstream_Rank18_F	TCACCCTGACCAGTGAAAAA
Off-target_downstream_Rank18_R	AATGGTGCTTGGATTGGAAG
Off-target_downstream_Rank19_F	TGTTTGATTGCTGCTGCTCT
Off-target_downstream_Rank19_R	TGCCAAGAAATGGAGAAACC
Off-target_downstream_Rank20_F	TTTGACTCTAAGCCCCCAGA
Off-target_downstream_Rank20_R	AGCTGCAGGAGAAAGAGGTG
Off-target_downstream_Rank21_F	AGGTTGGCATGGACTACAGG
Off-target_downstream_Rank21_R	TGCATGTGCTCACTGTCTGA
Off-target_downstream_Rank22_F	CTTCATGGGGAGCAGTGTTT
Off-target_downstream_Rank22_R	CTCCAGGTGTATGGCATGAA
Off-target_downstream_Rank23_F	AGGTTGGCATGGACTACAGG
Off-target_downstream_Rank23_R	TGCATGTGCTCACTGTCTGA
Off-target_downstream_Rank24_F	AGGTTTCCTGCAAAGCTTGA
Off-target_downstream_Rank24_R	AAAGCAATGTGTGGTGGTTTC
Off-target_downstream_Rank25_F	GGACTTGCAGAGCAGGACTC
Off-target_downstream_Rank25_R	CCACGTGCCTGTTCATGTAT
Off-target_downstream_Rank26_F	TGTGTGCAGGTGTGTGAAGA
Off-target_downstream_Rank26_R	TCCCCTACTGATCCGAATGT

Supplementary Table 2. The list of antibodies

Antibodies	Company
Rat anti-mouse CD3ε FITC (Clone 145-2C11)	BioLegend
Rat anti-mouse CD3ε PE (Clone 145-2C11)	BioLegend
Rat anti-mouse CD3ε PerCP/Cyanine5.5 (Clone 145-2C11)	BioLegend
Rat anti-mouse CD3ε Brilliant Violet 510 (Clone 145-2C11)	BioLegend
Rat anti-mouse CD4 FITC (Clone RM4-5)	BioLegend
Rat anti-mouse CD4 PE (Clone RM4-5)	BioLegend
Rat anti-mouse CD4 PE/Cyanine7 (Clone RM4-5)	BioLegend
Rat anti-mouse CD4 Brilliant Violet 510 (Clone RM4-5)	BioLegend
Rat anti-mouse CD5 FITC (Clone 53-7.3)	BioLegend
Rat anti-mouse CD5 PerCP/Cyanine5.5 (Clone 53-7.3)	BioLegend
Rat anti-Mouse CD8a FITC (Clone 53-6.7)	BD Bioscience
Rat anti-Mouse CD8a PE (Clone 53-6.7)	BD Bioscience
Rat anti-Mouse CD8a PE/Cyanine7 (Clone 53-6.7)	BioLegend
Rat anti-mouse/human CD11b FITC (Clone M1/70)	BioLegend
Rat anti-mouse/human CD11b Pe/Cyanine7 (Clone M1/70)	BioLegend
Hamster anti-mouse CD11c FITC (Clone N418)	BioLegend
Hamster anti-mouse CD11c PE/Cyanine7 (Clone N418)	BioLegend
Purified anti-mouse CD16/32 (Clone 93)	BioLegend
Rat anti-mouse CD19 FITC (Clone 6D5)	BioLegend
Rat anti-mouse CD25 PE (Clone PC61)	BioLegend
Rat anti-mouse CD25 Brilliant Violet 421 (Clone PC61)	BioLegend
Rat anti-mouse CD25 Brilliant Violet 510 (Clone PC61)	BioLegend
Rat anti-mouse CD44 PerCP-Cy5.5 (Clone IM7)	BD Bioscience
Rat anti-mouse/human CD45R/B220 FITC (Clone RA3-6B2)	BioLegend
Rat anti-mouse/human CD45R/B220 Brilliant Violet (Clone RA3-6B2)	BioLegend
Rat anti-mouse CD45 PerCP/Cyanine5.5 (Clone 30-F11)	BioLegend
Rat anti-mouse CD45 BV421 (Clone 30-F11)	BD Bioscience
Mouse anti-mouse CD45.1 FITC (Clone A20)	BioLegend
Mouse anti-mouse CD45.1 PerCP/Cyanine5.5 (Clone A20)	BioLegend
Mouse anti-mouse CD45.1 APC (Clone A20)	BioLegend
Mouse anti-mouse CD45.1 Brilliant Violet 605 (Clone A20)	BioLegend
Mouse anti-mouse CD45.2 APC/Cyanine7 (Clone 104)	BioLegend
Mouse anti-mouse CD45.2 Brilliant Violet 510 (Clone 104)	BioLegend
Mouse anti-mouse CD45.2 Brilliant Violet 785 (Clone 104)	BioLegend
Hamster anti-mouse CD49a APC (Clone HMα1)	BioLegend
Rat anti-mouse CD49b FITC (Clone DX5)	BioLegend
Rat anti-mouse CD49b PE (Clone DX5)	BioLegend
Rat anti-mouse CD62L PE (Clone MEL-14)	Thermo Fisher Scientific
Rat anti-mouse CD90.2 PerCP/Cyanine5.5 (Clone 53-2.1)	BioLegend
Rat anti-mouse CD90.2 PE/Cyanine7 (Clone 53-2.1)	BioLegend
Rat anti-mouse CD90.2 Brilliant Violet 510 (Clone 53-2.1)	BioLegend
Rat anti-mouse CD103 PE/Cyanine7 (Clone 2E7)	BioLegend
Rat anti-mouse CD127 PE/Cyanine7 (Clone A7R34)	BioLegend
Rat anti-mouse CD127 BV421 (Clone SB/199)	BD Bioscience
Mouse anti-mouse CD135 PerCP-eFluor 710 (Clone A2F10)	Thermo Fisher Scientific
Rat anti-mouse CD135 biotin (Clone A2F10)	BioLegend
Hamster anti-mouse CD196 (CCR6) PE (Clone 29-2L17)	BioLegend
Hamster anti-mouse CD196 (CCR6) APC (Clone 29-2L17)	BioLegend
Rat anti-mouse CD218a (IL-18Rα) PE (Clone A17071D)	BioLegend
Mouse anti-human CD271(NGFR) PerCP-Cy5.5 (Clone C40-1457)	BD Bioscience

Mouse anti-human CD271 (NGFR) V450 (Clone C40-1457)	BD Bioscience
Hamster anti-human/mouse/rat CD278 (ICOS) Brilliant Violet 421 (Clone C398.4A)	BioLegend
Rat anti-mouse CD279 PE/Cyanine7 (Clone 29F.1A12)	BioLegend
Rat anti-mouse CD279 PerCP/Cyanine5.5 Antibody (Clone 29F.1A12)	BioLegend
Mouse anti-mouse CD279 APC/Cyanine7 (Clone EH12.2H7)	BioLegend
Rat anti-mouse CD335 FITC (Clone 29A1.4)	BioLegend
Mouse anti-mouse/human GATA-3 eFluor 660 (Clone TWAJ)	Thermo Fisher Scientific
Mouse anti-mouse/human GATA-3 PE/Cyanine7 (Clone TWAJ)	Thermo Fisher Scientific
Mouse anti-mouse GATA3 Alexa Fluor 647 (Clone L50-823)	BD Bioscience
Rat anti-mouse I-A/I-E PerCP/Cyanine5.5 (Clone M5/114.15.2)	BioLegend
Rat anti-mouse Ly6A/E (Sca-1) PE (Clone E13-161-7)	BD Bioscience
Rat anti-mouse Ly6A/E (Sca-1) APC (Clone E13-161-7)	BioLegend
Rat anti-mouse Ly-6G/Ly-6C FITC (Clone RB6-8C5)	BioLegend
Rat anti-mouse Ly-6G FITC (Clone 1A8)	BioLegend
Rat anti-mouse Ly-6G APC (Clone 1A8)	BioLegend
Rat anti-mouse LPAM-1 PE (Clone DATK32)	BioLegend
Rat anti-mouse LPAM-1 APC (Clone DATK32)	BioLegend
Rat anti-mouse LPAM-1 PerCP-eFluor 710 (Clone DATK32)	Thermo Fisher Scientific
Hamster anti-mouse/human KLRG1 (MAFA) PE (Clone 2F1/KLRG1)	BioLegend
Hamster anti-mouse/human KLRG1 (MAFA) PE/Cyanine7 (Clone 2F1/KLRG1)	BioLegend
Rat anti-mouse/human IL-5 PE (Clone TRFK5)	BioLegend
Rat anti-mouse IL-13 eFluor 660 (Clone eBio13A)	Thermo Fisher Scientific
Rat anti-mouse IL-17RB Alexa Fluor 647 (Clone 9B10)	BioLegend
Rat anti-mouse IL-17RB PE (Clone 9B10)	BioLegend
Rat anti-mouse IL-33Rα (IL1RL1, ST2) PE/Cyanine7 (Clone DIH9)	BioLegend
Rat anti-mouse IL-33Rα (IL1RL1, ST2) APC (Clone DIH9)	BioLegend
Rat anti-mouse IL-33Rα (IL1RL1, ST2) Brilliant Violet 421 (Clone DIH9)	BioLegend
Hamster anti-mouse PLZF Antibody PE (Clone 9E12)	BioLegend
Mouse anti-mouse NK-1.1 (Clone PK136)	BioLegend
Mouse anti-mouse RORγt PE (Clone Q31-378)	BD Bioscience
Mouse anti-mouse RORγt BV421 (Clone Q31-378)	BD Bioscience
Mouse anti-mouse RORγt BV510 (Clone Q31-378)	BD Bioscience
Rat anti-mouse Siglec-F PE (Clone E50-2440)	BD Bioscience
Mouse anti-mouse/human T-bet PE (Clone 4B10)	BioLegend
Mouse anti-mouse/human T-bet PE/Cyanine7 (Clone 4B10)	BioLegend
Hamster anti-mouse TCR β chain FITC (Clone H57-597)	BioLegend
Hamster anti-mouse TCR γ/δ PE (Clone UC7-13D5)	BioLegend
Rat anti-mouse TER-119 FITC (Clone TER-119)	BioLegend
TotalSeq-A0302 anti-mouse Hashtag 2	BioLegend
TotalSeq-A0303 anti-mouse Hashtag 3	BioLegend
TotalSeq-A0307 anti-mouse Hashtag 7	BioLegend
TotalSeq-A0308 anti-mouse Hashtag 8	BioLegend
TotalSeq-A0309 anti-mouse Hashtag 9	BioLegend
TotalSeq-A0310 anti-mouse Hashtag 10	BioLegend
TotalSeq-A0311 anti-mouse Hashtag 11	BioLegend
TotalSeq-A0312 anti-mouse Hashtag 12	BioLegend
Anti-mouse CD3ε antibody (Clone 145-2C11)	BioXCell
Anti-mouse CD28 antibody (Clone 37.51)	BioXCell
Anti-mouse IFNγ antibody (Clone XMG1.2)	BioXCell
Anti-mouse IL-4 antibody (Clone 11B11)	BioXCell
Anti-H3K27ac antibody (Clone ab4729)	abcam
