# Supplementary material for: Stage-specific GATA3 induction promotes ILC2 development after lineage commitment
Source: Nat Commun. 2024 Jul 5;15:5610. doi: 10.1038/s41467-024-49881-y (PMC11226602; doi:10.1038/s41467-024-49881-y)
Supplement: Supplementary file 3 — Reporting Summary [file 41467_2024_49881_MOESM3_ESM.pdf]

nature portfolio

Corresponding author(s): Arifumi Iwata, Hiroshi Nakajima

Last updated by author(s): May 13, 2024

Reporting Summary

Nature Portfolio wishes to improve the reproducibility of the work that we publish. This form provides structure for consistency and transparency in reporting. For further information on Nature Portfolio policies, see our Editorial Policies and the Editorial Policy Checklist.

Statistics

For all statistical analyses, confirm that the following items are present in the figure legend, table legend, main text, or Methods section.

n/a	Confirmed	
☐	☒	The exact sample size (*n*) for each experimental group/condition, given as a discrete number and unit of measurement
☐	☒	A statement on whether measurements were taken from distinct samples or whether the same sample was measured repeatedly
☐	☒	The statistical test(s) used AND whether they are one- or two-sided<br>*Only common tests should be described solely by name; describe more complex techniques in the Methods section.*
☒	☐	A description of all covariates tested
☒	☐	A description of any assumptions or corrections, such as tests of normality and adjustment for multiple comparisons
☐	☒	A full description of the statistical parameters including central tendency (e.g. means) or other basic estimates (e.g. regression coefficient) AND variation (e.g. standard deviation) or associated estimates of uncertainty (e.g. confidence intervals)
☐	☒	For null hypothesis testing, the test statistic (e.g. *F*, *t*, *r*) with confidence intervals, effect sizes, degrees of freedom and *P* value noted<br>*Give P values as exact values whenever suitable.*
☒	☐	For Bayesian analysis, information on the choice of priors and Markov chain Monte Carlo settings
☒	☐	For hierarchical and complex designs, identification of the appropriate level for tests and full reporting of outcomes
☒	☐	Estimates of effect sizes (e.g. Cohen's *d*, Pearson's *r*), indicating how they were calculated

Our web collection on statistics for biologists contains articles on many of the points above.

Software and code

Policy information about availability of computer code

Data collection	BD FACSCantII, BD LSRFortessa X-20 were used for flow cytometry data collection. SONY SH800, BD FACSMelody and BD FACSAriaII were used for cell sorting. Illumina HiSeq2500 and NextSeq 500 were used for ChIP-seq and ATAC-seq data collection. BD Rhapsody and NovaSeq 6000 were used for TAS-seq (scRNA-seq).
Data analysis	FlowJo v10.7.1 and v10.8.1. were used for all flow data analysis. Weltch's T test was performed using R (v4.2.3) and RStudio (v2022.07.1). ANOVA was performed using lsmeans (2.30-0). ChIP-seq and ATAC-seq data were analyzed using SeqPurge (2019_09), Bowtie2 (v2.4.2), bedtools (2.30.0), edgeR (3.38.1), Homer (v4.11) and UCSC genome browser. scRNA-seq data was analyzed using Cutadapt (v3.4), Seurat (4.3.0), tidyverse(1.3.1), sctransform (03.5), slingshot (v2.4.0), tradeSeq (v1.10.0), qvalue (v2.28.0), clusterProfiler (v4.4.4), enrichplot (v1.16.1) and SingleCellExperiment (v1.18.0).

For manuscripts utilizing custom algorithms or software that are central to the research but not yet described in published literature, software must be made available to editors and reviewers. We strongly encourage code deposition in a community repository (e.g. GitHub). See the Nature Portfolio guidelines for submitting code & software for further information.

Data

Policy information about availability of data

All manuscripts must include a data availability statement. This statement should provide the following information, where applicable:

- Accession codes, unique identifiers, or web links for publicly available datasets
- A description of any restrictions on data availability
- For clinical datasets or third party data, please ensure that the statement adheres to our policy

All sequencing data generated in this study have been deposited in the GEO database under accession code GSE218144 [https://www.ncbi.nlm.nih.gov/geo/query/acc.cgi?acc=GSE218144] for ChIP-seq, GSE218142 [https://www.ncbi.nlm.nih.gov/geo/query/acc.cgi?acc=GSE218142] for ATAC-seq, and GSE218144 [https://www.ncbi.nlm.nih.gov/geo/query/acc.cgi?acc=GSE218154] for scRNA-seq. Published ATAC-seq data of cultured helper T cell subsets were from GSE15950531 [https://www.ncbi.nlm.nih.gov/geo/query/acc.cgi?acc=GSE159505]. Published ChIP-seq data for GATA3 and Bcl11b were from GSE11187137 [https://www.ncbi.nlm.nih.gov/geo/query/acc.cgi?acc=GSE111871] and GSE13108256 [https://www.ncbi.nlm.nih.gov/geo/query/acc.cgi?acc=GSE131082], respectively.

Research involving human participants, their data, or biological material

Policy information about studies with human participants or human data. See also policy information about sex, gender (identity/presentation), and sexual orientation and race, ethnicity and racism.

Reporting on sex and gender	N/A
Reporting on race, ethnicity, or other socially relevant groupings	N/A
Population characteristics	N/A
Recruitment	N/A
Ethics oversight	N/A

Note that full information on the approval of the study protocol must also be provided in the manuscript.

Field-specific reporting

Please select the one below that is the best fit for your research. If you are not sure, read the appropriate sections before making your selection.

[x] Life sciences [ ] Behavioural & social sciences [ ] Ecological, evolutionary & environmental sciences

For a reference copy of the document with all sections, see nature.com/documents/nr-reporting-summary-flat.pdf

Life sciences study design

All studies must disclose on these points even when the disclosure is negative.

Sample size	Sample sizes were estimated based on our previous studies. For in vivo experiments, 4-7 mice per group were used. For in vitro culture experiments, 3-5 mice per group were used. Sample sizes were described in the figure legends.
Data exclusions	No data were excluded.
Replication	Reproducibility were ensured at least two independent experiments. Repeated numbers of experiments were described in the figure legends.
Randomization	No randomization was performed. Age- and sex-matched littermates were used as control.
Blinding	No blinding was performed.

Reporting for specific materials, systems and methods

We require information from authors about some types of materials, experimental systems and methods used in many studies. Here, indicate whether each material, system or method listed is relevant to your study. If you are not sure if a list item applies to your research, read the appropriate section before selecting a response.

Materials & experimental systems

n/a	Involved in the study
	☒ Antibodies
	☒ Eukaryotic cell lines
☒	Palaeontology and archaeology
	☒ Animals and other organisms
☒	Clinical data
☒	Dual use research of concern
☒	Plants

Methods

n/a	Involved in the study
	☒ ChIP-seq
	☒ Flow cytometry
☒	MRI-based neuroimaging

Antibodies

Antibodies used

For flow cytometry analysis:
FITC anti-mouse CD3e Antibody (BioLegend, Cat# 100305, Clone 145-2C11, 1:100 dilution, RRID: AB_312670)
PE anti-mouse CD3e Antibody (BioLegend, Cat# 100308, Clone 145-2C11, 1:100 dilution, RRID: AB_312673)
PerCP/Cyanine5.5 anti-mouse CD3e Antibody (BioLegend, Cat# 100328, Clone 145-2C11, 1:100 dilution, RRID: AB_893318)
Brilliant Violet 510 anti-mouse CD3e Antibody (BioLegend, Cat# 100353, Clone 145-2C11, 1:100 dilution, RRID: AB_2565879)
FITC anti-mouse CD4 Antibody (BioLegend, Cat# 100510, Clone RM4-5, 1:100 dilution, RRID: AB_312713)
PE anti-mouse CD4 Antibody (BioLegend, Cat# 100512, Clone RM4-5, 1:100 dilution, RRID: AB_312715)
PE/Cyanine 7 anti-mouse CD4 Antibody (BioLegend, Cat# 100528, Clone RM4-5, 1:100 dilution, RRID: AB_312729)
Brilliant Violet 510 anti-mouse CD4 Antibody (BioLegend, Cat# 100559, Clone RM4-5, 1:100 dilution, RRID: AB_2562608)
FITC anti-mouse CD5 Antibody (Biolegend, Cat# 100606, Clone 53-7.3, 1:100 dilution, RRID: AB_312735)
PerCP/Cyanine5.5 anti-mouse CD5 Antibody (BioLegend, Cat# 100624, Clone 53-7.3, 1:100 dilution, RRID: AB_2563433)
FITC anti-mouse CD8a Antibody (BD Bioscience, Cat# 553031, Clone 53-6.7, 1:100 dilution, RRID: AB_394569)
PE anti-mouse CD8a Antibody (BD Bioscience, Cat# 553033, Clone 53-6.7, 1:100 dilution, RRID: AB_394571)
PE/Cyanine7 anti-mouse CD8a Antibody (Biolegend, Cat# 100722, Clone 53-6.7, 1:100 dilution, RRID: AB_312761)
FITC anti-mouse/human CD11b Antibody (Biolegend, Cat# 101206, Clone M1/70, 1:50 dilution, RRID: AB_312789)
PE/Cyanine7 anti-mouse/human CD11b Antibody (Biolegend, Cat# 101216, Clone M1/70, 1:50 dilution, RRID: AB_312799)
FITC anti-mouse CD11c Antibody (Biolegend, Cat# 117306, Clone N418, 1:50 dilution, RRID: AB_313775)
PE/Cyanin7 anti-mouse CD11c Antibody (BioLegend, Cat# 117317, Clone N418, 1:50 dilution, RRID: AB_493569)
Purified anti-mouse CD16/32 Antibody (BioLegend, Cat# 101302, Clone 93, 1:100 dilution, RRID: AB_312801)
FITC anti-mouse CD19 Antibody (BioLegend, Cat# 115506, Clone 6D5, 1:50 dilution, RRID: AB_313641)
PE anti-mouse CD25 Antibody (BioLegend, Cat# 102008, Clone PC61, 1:100 dilution, RRID: AB_312857)
Brilliant Violet 421 anti-mouse CD25 Antibody (BioLegend, Cat# 102034, Clone PC61, 1:100 dilution, RRID: AB_11203373)
Brilliant Violet 510 anti-mouse CD25 Antibody (BioLegend, Cat# 102042, Clone PC61, 1:100 dilution, RRID: AB_2562270)
PerCP-Cy5.5 anti-mouse CD44 Antibody (BD Bioscience, Cat# 560570, Clone IM7, 1:100 dilution, RRID: AB_1727486)
FITC anti-mouse/human CD45R/B220 Antibody (BioLegend, Cat# 103206, Clone RA3-6B2, 1:50 dilution, RRID: AB_312991)
Brilliant Violet 421 anti-mouse/human CD45R/B220 Antibody (BioLegend, Cat# 103240, Clone RA3-6B2, 1:50 dilution, RRID: AB_11203896)
PerCP/Cyanine5.5 anti-mouse CD45 Antibody (BioLegend, Cat# 103132, Clone 30-F11, 1:200 dilution, RRID: AB_893340)
BV421 anti-mouse CD45 Antibody (BD Bioscience, Cat# 563890, Clone 30-F11, 1:200 dilution, RRID: AB_2651151)
FITC anti-mouse CD45.1 Antibody (BioLegend, Cat# 110706, Clone A20, 1:200 dilution, RRID: AB_313495)
PerCP/Cyanine5.5 anti-mouse CD45.1 Antibody (BioLegend, Cat# 110727, Clone A20, 1:200 dilution, RRID: AB_893348)
APC anti-mouse CD45.1 Antibody (BioLegend, Cat# 110714, Clone A20, 1:200 dilution, RRID: AB_313503)
Brilliant Violet 605 anti-mouse CD45.1 Antibody (BioLegend, Cat# 110738, Clone A20, 1:200 dilution, RRID: AB_2562565)
APC/Cyanine7 anti-mouse CD45.2 Antibody (BioLegend, Cat# 109824, Clone 104, 1:200 dilution, RRID: AB_830789)
Brilliant Violet 510 anti-mouse CD45.2 Antibody (BioLegend, Cat# 109837, Clone 104, 1:200 dilution, RRID: AB_2561393)
Brilliant Violet 785 anti-mouse CD45.2 Antibody (BioLegend, Cat# 109837, Clone 104, 1:200 dilution, RRID: AB_2561393)
APC anti-mouse CD49a APC Antibody (BioLegend, Cat# 142606, Clone HMα1, 1:100 dilution, RRID: AB_2562253)
FITC anti-mouse CD49b Antibody (BioLegend, Cat# 108906, Clone DX5, 1:50 dilution, RRID: AB_313413)
PE anti-mouse CD49b Antibody (BioLegend, Cat# 108908, Clone DX5, 1:50 dilution, RRID: AB_313415)
PE anti-mouse CD62L Antibody (Thermo Fisher Scientific, Cat# 12-0621-83, Clone MEL-14, 1:100 dilution, RRID: AB_465722)
PerCP/Cyanine5.5 anti-mouse CD90.2 Antibody (BioLegend, Cat# 140322, Clone 53-2.1, 1:100 dilution, RRID: AB_2562696)
PE/Cyanine7 anti-mouse CD90.2 Antibody (BioLegend, Cat# 140310, Clone 53-2.1, 1:100 dilution, RRID: AB_10643586)
Brilliant Violet 510 anti-mouse CD90.2 Antibody (BioLegend, Cat# 140319, Clone 53-2.1, 1:100 dilution, RRID: AB_2561395)
PE/Cyanine7 anti-mouse CD103 Antibody (BioLegend, Cat# 121426, Clone 2E7, 1:100 dilution, RRID: AB_2563691)
PE/Cyanine7 anti-mouse CD127 Antibody (BioLegend, Cat# 135014, Clone A7R34, 1:50 dilution, RRID: AB_1937265)
BV421 anti-mouse CD127 Antibody (BD Bioscience, Cat# 562959, Clone SB/199, 1:50 dilution, RRID: AB_2737917)
PerCP-eFluor 710 anti-mouse CD135 (FLT3) Antibody (Thermo Fisher Scientific, Cat# 46-1351-82, Clone A2F10, 1:50 dilution, RRID: AB_10733393)
Biotin anti-mouse CD135 (FLT3) Antibody (BioLegend, Cat# 135308, Clone A2F10, 1:100 dilution, RRID: AB_1953267)
PE anti-mouse CD196 (CCR6) Antibody (BioLegend, Cat# 129804, Clone 29-2L17, 1:50 dilution, RRID: AB_1279137)
APC anti-mouse CD196 (CCR6) Antibody (BioLegend, Cat# 129814, Clone 29-2L17, 1:50 dilution, RRID: AB_1877147)
PE anti-mouse CD218a (IL-18Rα) Antibody (BioLegend, Cat# 157904, Clone A17071D, 1:100 dilution, RRID: AB_2860733)
PerCP-Cy5.5 anti-human CD271 (NGFR) Antibody (BD Bioscience, Cat# 560834, Clone C40-1457, 1:200 dilution, RRID: AB_10561839)

V450 anti-human CD271 (NGFR) Antibody (BD Bioscience, Cat# 562123, Clone A51:D980-1457, 1:200 dilution, RRID: AB_10926195)
Pacific Blue anti-human/mouse/rat CD278 (ICOS) Antibody (BioLegend, Cat# 313522, Clone C398.4A, 1:100 dilution, RRID: AB_10899736)
PE/Cyanine7 anti-mouse CD279 (PD-1) Antibody (BioLegend, Cat# 135216, Clone 29F.1A12, 1:100 dilution, RRID: AB_10689635)
PerCP/Cyanine5.5 anti-mouse CD279 (PD-1) Antibody (BioLegend, Cat# 135208, Clone 29F.1A12, 1:100 dilution, RRID: AB_2159184)
APC/Cyanine7 anti-mouse CD279 (PD-1) Antibody (BioLegend, Cat# 329922, Clone EH12.2H7, 1:100 dilution, RRID: AB_10933429)
FITC anti-mouse CD335 (NKp46) Antibody (BioLegend, Cat# 137605, Clone 29A1.4, 1:100 dilution, RRID: AB_2149150)
eFluor 660 anti-mouse/human GATA-3 Antibody (Thermo Fisher Scientific, Cat# 50-9966-42, Clone TWAJ, 1:50 dilution, RRID: AB_10596663)
PE/Cyanine7 anti-mouse/human GATA-3 Antibody (Thermo Fisher Scientific, Cat# 25-9966-42, Clone TWAJ, 1:50 dilution, RRID: AB_2573568)
Alexa Fluor 647 anti-mouse GATA3 Antibody (BD Bioscience, Cat# 560078, Clone L50-823, 1:50 dilution, RRID: AB_1645317)
PerCP/Cyanine5.5 anti-mouse I-A/I-E Antibody (BioLegend, Cat# 107626, Clone M5/114.15.2, 1:200 dilution, RRID: AB_2191071)
PE anti-mouse Ly6A/E (Sca1) Antibody (BD Bioscience, Cat# 553333, Clone E13-161-7, 1:200 dilution, RRID: AB_394789)
APC anti-mouse Ly6A/E (Sca1) Antibody (BioLegend, Cat# 122512, Clone E13-161-7, 1:200 dilution, RRID: AB_756197)
FITC anti-mouse Ly-6G/Ly-6C Antibody (BioLegend, Cat# 108405, Clone RB6-8C5, 1:50 dilution, RRID: AB_313370)
FITC anti-mouse Ly-6G Antibody (BioLegend, Cat# 127606, Clone 1A8, 1:100 dilution, RRID: AB_1236494)
APC anti-mouse Ly-6G Antibody (BioLegend, Cat# 127614, Clone 1A8, 1:100 dilution, RRID: AB_2227348)
PE anti-mouse Integrin α4β7 (LPAM) Antibody (BioLegend, Cat# 120606, Clone DATK32, 1:100 dilution, RRID: AB_493267)
APC anti-mouse Integrin α4β7 (LPAM) Antibody (BioLegend, Cat# 120608, Clone DATK32, 1:100 dilution, RRID: AB_10730607)
PerCP-eFluor 710 anti-mouse Integrin α4β7 (LPAM) Antibody (Thermo Fisher Scientific, Cat# 46-5887-82, Clone DATK32, 1:100 dilution, RRID: AB_2573793)
PE anti-mouse/human KLRG1 (MAFA) Antibody (BioLegend, Cat# 138408, Clone 2F1/KLRG1, 1:100 dilution, RRID: AB_10574313)
PE/Cyanine7 anti-mouse/human KLRG1 (MAFA) Antibody (BioLegend, Cat# 138416, Clone 2F1/KLRG1, 1:100 dilution, RRID: AB_2561736)
PE anti-mouse/human IL-5 Antibody (BioLegend, Cat# 504304, Clone TRFK5, 1:100 dilution, RRID: AB_2574279)
eFluor660 anti-mouse IL-13 Antibody (Thermo Fisher Scientific, Cat# 50-7133-82, Clone eBio13A, 1:100 dilution, RRID: AB_2574279)
Alexa Fluor 647 anti-mouse IL-17RB Antibody (BioLegend, Cat# 146304, Clone 9B10, 1:100 dilution, RRID: AB_2562381)
PE anti-mouse IL-17RB Antibody (BioLegend, Cat# 146306, Clone 9B10, 1:100 dilution, RRID: AB_2564512)
PE/Cyanine7 anti-mouse IL-33Rα (IL1RL1, ST2) Antibody (BioLegend, Cat# 145316, Clone DIH9, 1:50 dilution, RRID: AB_2687367)
APC anti-mouse IL-33Rα (IL1RL1, ST2) Antibody (BioLegend, Cat# 145306, Clone DIH9, 1:50 dilution, RRID: AB_2561917)
Brilliant Violet 421 anti-mouse IL-33Rα (IL1RL1, ST2) Antibody (BioLegend, Cat# 145309, Clone DIH9, 1:100 dilution, RRID: AB_2565634)
PE anti-mouse PLZF Antibody (BioLegend, Cat# 145804, Clone 9E12, 1:100 dilution, RRID: AB_2561973)
FITC anti-mouse NK1.1 Antibody (BioLegend, Cat# 108706, Clone PK136, 1:100, dilution, RRID: AB_3133393)
PE anti-mouse RORγt Antibody (BD Bioscience, Cat# 562607, Clone Q31-378, 1:100 dilution, RRID: AB_11153137)
BV421 anti-mouse RORγt Antibody (BD Bioscience, Cat# 562894, Clone Q31-378, 1:100 dilution, RRID: AB_2687545)
BV510 anti-mouse RORγt Antibody (BD Bioscience, Cat# 567177, Clone Q31-378, 1:100 dilution, RRID: AB_2916491)
PE anti-mouse Siglec-F Antibody (BD Bioscience, Cat# 552126, Clone E50-2440, 1:100 dilution, RRID: AB_394341)
PE anti-mouse/human T-bet Antibody (BioLegend, Cat# 644810, Clone 4B10, 1:100 dilution, RRID: AB_2200542)
PE/Cyanine7 anti-mouse/human T-bet Antibody (BioLegend, Cat# 644824, Clone 4B10, 1:100 dilution, RRID: AB_2561761)
FITC anti-mouse TCR β chain Antibody (BioLegend, Cat# 109206, Clone H57-597, 1:100 dilution, RRID: AB_313429)
PE anti-mouse TCR γ/δ Antibody (BioLegend, Cat# 107508, Clone UC7-13D5, 1:100 dilution, RRID: AB_345266)
FITC anti-mouse Ter119 Antibody (BioLegend, Cat# 116206, Clone TER-119, 1:50 dilution, RRID: AB_313707)

For in vivo experiments:
Anti-mouse CD3e Antibody (BioXCell, Cat# BE0001-1, Clone 145-2C11, RRID: AB_1107634)
Anti-CD28 Antibody (BioXCell, Cat# BE0015-1, Clone 37.51, RRID: AB_1107624)
Anti-IFNg Antibody (BioXCell, Cat# BP0055, Clone XMG1.2, RRID: AB_1107694)
Anti-IL-4 Antibody (BioXCell, Cat# BP0045, Clone 11B11, RRID: AB_1107707)

For ChIP-seq analysis:
Anti-H3K27ac Antibody (Abcam, Cat# ab4729, Clone ab4729, RRID: AB_2118291)

For single-cell RNA-sequencing analysis:
Hashtag 2 anti-mouse TotalSeq-A0302 (BioLegend, Cat# 155803, RRID: AB_2750033)
Hashtag 3 anti-mouse TotalSeq-A0303 (BioLegend, Cat# 155805, RRID: AB_2750034)
Hashtag 7 anti-mouse TotalSeq-A0307 (BioLegend, Cat# 155813, RRID: AB_2750039)
Hashtag 8 anti-mouse TotalSeq-A0308 (BioLegend, Cat# 155815, RRID: AB_2750040)
Hashtag 9 anti-mouse TotalSeq-A0309 (BioLegend, Cat# 155817, RRID: AB_2750042)
Hashtag 10 anti-mouse TotalSeq-A0310 (BioLegend, Cat# 155819, RRID: AB_2750043)
Hashtag 11 anti-mouse TotalSeq-A0311 (BioLegend, Cat# 155821, RRID: AB_2750136)
Hashtag 12 anti-mouse TotalSeq-A0312 (BioLegend, Cat# 155823, RRID: AB_2750137)

Validation

For the validation information, we refer to the information provided by the supplying companies.

Eukaryotic cell lines

Policy information about cell lines and Sex and Gender in Research

Cell line source(s)	Plat-E cells were provided by Dr. Daisuke Kitamura (Tokyo University of Science).<br>OP9-DL1 cells were provided by Dr. Hiroyuki Hosokawa (Tokai University School of Medicine).
Authentication	The cell lines were not authenticated.
Mycoplasma contamination	Mycoplasma test were negative.
Commonly misidentified lines<br>(See ICLAC register)	No commonly misidentified lines were used in this study.

Animals and other research organisms

Policy information about studies involving animals; ARRIVE guidelines recommended for reporting animal research, and Sex and Gender in Research

Laboratory animals	C57BL/6J were purchased from CLEA Japan Inc. (Tokyo, Japan), and CD45.1 mice were purchased from RIKEN BRC (Ibaraki, Japan). The G3SEKO mice were generated using CRIPSR-Cas9 system in Biomedeical Resewarch Center, Chiba University. Mice were housed in micro-isolator cages under specific pathogen-free conditions. Six- to twelve-week-old male and female mice were used in this study.
Wild animals	No wild animals were used in this study.
Reporting on sex	We used both sex mice for in vivo steady state experiments and in vitro experiments. We used female mice for the asthma model as it is known that phenotype is attenuated in male mice.
Field-collected samples	No field-collected samples were used in this study
Ethics oversight	Chiba University Animal Care and Use Committee approved the animal procedures used in this study.

Note that full information on the approval of the study protocol must also be provided in the manuscript.

Plants

Seed stocks	N/A
Novel plant genotypes	N/A
Authentication	N/A

ChIP-seq

Data deposition

☒ Confirm that both raw and final processed data have been deposited in a public database such as GEO.

☒ Confirm that you have deposited or provided access to graph files (e.g. BED files) for the called peaks.

Data access links<br>*May remain private before publication.*	To review GEO accession GSE218144:<br>Go to https://www.ncbi.nlm.nih.gov/geo/query/acc.cgi?acc=GSE218144
Files in database submission	GSM6735425_DK18117_15.fastq.gz<br>GSM6735426_DK18098_12.fastq.gz<br>GSM6735427_DK18117_7.fastq.gz<br>GSM6735428_DK18098_14.fastq.gz<br>GSM6735425_ChIP-seq_H3K27ac_CD4T_control.bedgraph.gz<br>GSM6735426_ChIP-seq_H3K27ac_CD4T_asthma.bedgraph.gz<br>GSM6735427_ChIP-seq_H3K27ac_ILC2_control.bedgraph.gz<br>GSM6735428_ChIP-seq_H3K27ac_ILC2_asthma.bedgraph.gz
Genome browser session<br>(e.g. UCSC)	*Provide a link to an anonymized genome browser session for "Initial submission" and "Revised version" documents only, to enable peer review. Write "no longer applicable" for "Final submission" documents.*

Methodology

Replicates	No replicates for ChIP-seq in this study.

Sequencing depth	Sequencing were perform using HiSeq2500 with single-end 50bp. More than forty million reads were obtained from each sample. Ninety percent of reads were uniquely mapped on mouse mm10 genome.
Antibodies	abcam Rabbit polyclonal antibody to anti-Histone H3 (acetyl K27) - ChIP Grade (cat # ab4729).
Peak calling parameters	SEs from H3K27ac ChIP-seq data were identified using findPeaks in the HOMER package with the following parameter, -style superhistone, -poisson 1e-8, and -excludePeaks option for excluding TSS ± 2500 kb region.
Data quality	All ChIP-seq dataset exhibited more than 30000 peaks above the threshold FDR 5%. Appropriate IP efficiency is more than 20%. Analysis in this paper employed "-poisson 1e-8" as the threshold.
Software	ChIP-seq data was analyzed using Bowtie2 (v2.4.2), edgeR (3.38.1), Homer (v4.11) and UCSC genome browser.

Flow Cytometry

Plots

Confirm that:

☒ The axis labels state the marker and fluorochrome used (e.g. CD4-FITC).

☒ The axis scales are clearly visible. Include numbers along axes only for bottom left plot of group (a 'group' is an analysis of identical markers).

☒ All plots are contour plots with outliers or pseudocolor plots.

☒ A numerical value for number of cells or percentage (with statistics) is provided.

Methodology

Sample preparation	Single-cell suspensions of the lungs were generated as below. Lungs were cut into small pieces and digested in RPMI containing Collagenase A (1 mg/ml) and DNase I (0.1 mg/ml) using gentleMACS Dissociator (Miltenyi Biotec) for 30 min at 37ºC and then passed through 70 µm filter.<br>Single-cell suspensions of the bone marrow cells were harvested from the cut-off of both ends of the femur, tibia and pelvis, humerus, and scapula of mice using a centrifuge.<br>Splenocytes and thymocytes were isolated by physical dissociation.<br>Single-cell suspensions of the liver cells were prepared as below. The liver was minced into small pieces after perfusion with 10 ml PBS and digested in RPMI containing Liberase TM (0.05 mg/ml) and DNase I (0.2 mg/ml) for 45 min at 37ºC using gentleMACS Dissociator. After passing through a 100 µm filter, hepatic leukocytes were isolated from the interface between 40% and 80% Percoll gradient.<br>Single-cell suspensions of the small intestine cells were gererated as below. The small intestine was opened longitudinally and washed four times with PBS. After cutting the small intestine into pieces, mucus and epithelial cells were removed by first incubating 15 min at 37ºC in HBSS containing 10% FBS, 15m M HEPES, 5 mM EDTA, and 1 mM DTT. After washing thoroughly with PBS three times, the samples were minced into small pieces and digested in RPM containing 10% FBS, Liberase TL (0.25 mg/ml), and DNase I (0.3 mg/ml) for 30 min at 37ºC using gentleMACS Dissociator. After passing through a 100 µm filter, intestine leukocytes were purified using the Percoll gradient as described above.<br>All single cells were passed through a 30 µm filter before downstream experiments.
Instrument	BD FACSCantII, BD LSRFortessa X-20 were used for flow cytometry data collection. SONY SH800, BD FACSMelody and BD FACSAriaII were used for cell sorting.
Software	FlowJo Version 10.7.1 and 10.8.1.
Cell population abundance	Post sort purity was more than 90%.
Gating strategy	The lymphocytes were gated based on FSC-A and SSC-A. Singlets were gated based on FSC-A and FSC-W. Live cells were identified as Zombie-dye negative cells. Cell specific gating strategy were described in Supplementary Fig. 4.

☒ Tick this box to confirm that a figure exemplifying the gating strategy is provided in the Supplementary Information.
